# Concordance of MRI-Guided Fusion and Systematic 12-Core Prostate Biopsy for the Detection of Prostate Cancer

**DOI:** 10.3389/fonc.2022.899567

**Published:** 2022-05-27

**Authors:** Matthew Parsons, Zoya Sandhu, Bridget Foy, Ernest Chan, Bryan Crawford, Libby Petersen, Benjamin Romney, Daniel Sommers, Jay Bishoff, Steven Lynch, Logan Mclean, David Gill

**Affiliations:** ^1^ Department of Radiation Oncology, Huntsman Cancer Institute, University of Utah, Salt Lake City, UT, United States; ^2^ College of Osteopathic Medicine, Rocky Vista University, Ivins, UT, United States; ^3^ Intermountain Medical Center, Murray, UT, United States

**Keywords:** prostate cancer, biopsy - methods, MRI guidance, quality improvement, 12 core biopsy

## Abstract

**Background:**

MRI-guided fusion biopsy is increasingly utilized over systematic 12-core biopsy for men with MRI-visible prostate lesions.

**Patients and Methods:**

Patients with MRI visible lesions who underwent MRI-guided fusion and systematic 12-core biopsy from 2016-2020 in the Intermountain Healthcare (IHC) system were consecutively analyzed. This was in the setting of a continuous quality assurance initiative among the reading radiologists. Primary outcome was prostate cancer (PCa) detection defined by Gleason grade group (GGG) 1 or higher. Clinically significant cancer (CSC) was defined as GGG 2 or higher. Patients were stratified by biopsy date, 2016-2017 and 2018-2021, and lesions were stratified by PI-RADS v2 category.

**Results:**

A total of 184 patients with 324 MRI-detectable lesions underwent both biopsy modalities in the IHC system from 2016 to 2021. CSC was detected in 23.5% of MRI-guided fusion biopsies. Comparing PI-RAD v2 categories 1-3 to categories 4-5, rate of CSC was 10% and 42% respectively. MRI-guided fusion and systematic 12-core biopsies were concordant for PCa in 77% of men and CSC in 83%. MRI-guided fusion biopsy detected PCa in 26/103 and CSC in 20/131 men in whom systematic 12-core biopsy was negative. Systematic 12-core biopsy detected PCa in 17/94 and CSC in 11/122 men in whom MRI-guided fusion was negative.

**Conclusions:**

Omitting MRI-guided fusion or systematic 12-core biopsy would have resulted in underdiagnosis of CSC in 11% or 6% of patients respectively. Combining biopsies increased detection rate of CSC. This was in the setting of a continuous quality assurance program at a large community-based hospital.

## Introduction

Prostate cancer (PCa) is the most commonly diagnosed cancer in men globally with 191,930 new cases in the United States in 2020 and an estimate of 3,170,339 men living with prostate cancer in 2017 (1, 2). However, the management and outcomes of patients with PCa vary widely by risk group (3, 4). After publication of the prostate, lung, colorectal, and ovarian (PLCO) (5) and European Randomized Study of Screening for Prostate Cancer (ERSPC) (6) trials, which showed a questionable benefit for PSA screening and a high risk of overdiagnosis, the United States Preventative Services Task Force (USPSTF) has recommended against population based PCa screening (7). This has raised awareness of the importance of active surveillance for National Comprehensive Cancer Network (NCCN) low-risk PCa. This is defined as PCa with Gleason grade group (GGG) 1, prostate specific antigen (PSA) <10, and clinical stage <T2b (4). In addition, this highlights the need to differentiate between NCCN low-risk prostate cancer and clinically significant prostate cancer defined as GGG ≥2. Independent of NCCN risk grouping Gleason score has been shown to have prognostic value. Tumors with higher Gleason scores have a tendency to metastasize to areas outside of the prostate, leading to a poor clinical course and survival rate (8). Given the impact of Gleason score on treatment options and patient outcomes accurate tissue evaluation is an imperative part of the workup of PCa (9).

The USPSTF recommends trans-rectal ultrasonography (TRUS)-guided systematic 12-core biopsy to diagnose PCa (7). This procedure involves using ultrasound as a guide while taking 12 biopsies from 6 areas in the prostate. 12-core biopsies are systematic but can have limited sampling of the anterior gland. Developed to improve the accuracy of systematic 12-core biopsy, MRI-guided fusion biopsy utilizes MRI/US to visualize suspicious lesions within the gland and facilitates targeted biopsy (10, 11). Prostate Imaging - Reporting and Data System (PI-RADS) is a standardized guide to interpret prostate MRIs. PI-RADS provides assessment categories and establishes levels of risk and suspicion for clinically significant prostate cancer (CSC) with PI-RADS score 4 and 5 representing high risk for CSC (12).

Recent studies support improved CSC detection rate with MRI-guided fusion biopsies compared to traditional 12-core biopsy approach. However, some data argues against completely replacing systematic 12-core biopsies. A recent analysis found that MRI-guided fusion biopsies alone misclassified up to 8.8% of CSCs and that pathology on radical prostatectomy (RP) was least likely to result in a upgrade in patients who underwent both forms of biopsy (13). Until now the preponderance of data on the question of MRI-guided fusion vs systematic 12-core biopsy has been from academic centers with patients on clinical trials. Therefore we set out to evaluate the performance of MRI-guided fusion and systematic 12-core biopsy in a continuous prospectively selected cohort at a large community-based hospital in the setting of an ongoing quality improvement initiative.

## Methods

### Study Population

Data for patients undergoing prostate MRI and MRI-guided fusion biopsy for the workup of a newly diagnosed prostate cancer in the Intermountain Healthcare system was collected. Data from 2016-2021 consisting of 184 consecutive patients with 324 identifiable and PI-RADS graded lesions were collected. All patients undergoing MRI-guided fusion biopsy during this time period also underwent systematic 12-core biopsy. Therefore, any patient who had was deemed by his treating physician to require MRI-guided biopsy was included in this study. There were no specific exclusion criteria, however patients with contraindications to MRI, such as medical implants would not have been included. Institutional review board approval was obtained for this study. Patient demographics and pre-biopsy PSA level, number of MRI lesions, and PI-RADS score per lesion were noted, as were final pathology for standard and targeted biopsies.

### Imaging

MRIs were reviewed by 3 fellowship trained genitourinary radiologists with more than 10 years of experience in reviewing prostate MRI. All MRIs were performed in compliance with imaging recommendations from the with ACR PI-RADS manual (version 1.0, then updated to version 2.1 2019).

MR lesions were scored using the scoring system from the PI-RADS manual. Before MRI-guided fusion biopsy, radiologists segmented the prostate gland and drew regions of interest for any identified lesions using the DynaCAD (Philips) software.

### Biopsy Protocol

All prostate biopsies were performed by a urologist with expertise in the technique of MRI-guided fusion biopsy. Fusion of MRI and US images were performed using the DynaCAD (Phillips) software. First, two or three biopsies were performed in each region of interest identified on MRI imaging. All MRI identified lesions were targeted regardless of PI-RADS score. Next, 12-core sextant biopsies were performed. The prostate was divided into six sextants and two biopsies were performed from each sextant. The sextants were defined as the base, mid, and apex of the prostate bilaterally.

### Quality Assurance

Patients with discordant MRI and MRI-guided fusion biopsy results were identified. Radiologists reviewed imaging and pathology of these instances. Discrepancies were defined as PI-RADS 4-5 lesions with negative MRI-guided fusion biopsy results (false positive MRI), or patients with PI-RADS 1-2 lesions and MRI-guided fusion biopsy results positive for cancer (false negative MRI). Discordant cases underwent joint review and were discussed by the 3 radiologists.

### Prostatectomy Cohort

Patients diagnosed with prostate cancer were offered various treatments, including active surveillance, prostatectomy, external beam radiation and brachytherapy. For patients who underwent RP, the surgical pathology was correlated with biopsy findings. Patients with an interval of greater than one year from biopsy to prostatectomy were excluded from this analysis

### Data Analysis

Patient data were retrospectively transferred from the electronic medical record into a REDcap online database. Reports were generated from REDcap containing patients MRI results and corresponding pathology from both MRI-guided fusion and systematic 12-core biopsy. Data was evaluated for sensitivity and specificity of MRI-guided fusion and systematic 12-core biopsy for any PCa and CSC as well as the positive predictive value for MRI identified lesions stratified by PI-RADS score.

## Results

A total of 184 men with 324 MRI-detectable lesions underwent both MRI-guided fusion and systematic 12-core biopsies in the IHC system from 2016 to 2021. The mean number of targetable lesions was 1.8 (range 1-4). In total 14% of identified lesions were PI-RADS 5, 27% PI-RADS 4, 33% PI-RADS 3, 22% PI-RADS 2 and 3% PI-RADS 1. Comparing MRIs performed 2015-2017 to those performed in 2018 and beyond, the proportion of high-grade lesions (PI-RADS 4-5) was significantly higher in the later cohort (28% vs 56%). PI-RADS 1-2 lesions were noted to be unlikely to correlate with CSC and as part of multidisciplinary quality improvement program oversight, the number of PI-RADS 1-2 lesions noted on prostate MRIs decreased dramatically from 2015-2017 compared to 2018-present (39% vs 11%). Further, highest PI-RADS score ≤2 occurred in 22/86 (26%) patients from 2015-2017 compared to 3/98 (3%) patients from 2018-present. Of 25 patients without PI-RADS lesions >2, only 2 (8%) had CSC (**Table 1**).

**Table 1:** Occurrence of any prostate cancer and clinically significant prostate cancer in MRI-fusion guided biopsy targets stratified by PI-RADS score and year of biopsy.

	2015-2017			2018-2021			All		
PI-RADS	Lesions	PCa (%)	CSC (%)	Lesions	PCa (%)	CSC (%)	Lesions	PCa (%)	CSC (%)
1	7	2 (29)	1 (14)	2	0 (0)	0 (0)	9	2 (22)	1 (11)
2	55	10 (18)	5 (9)	16	3 (19)	2 (13)	71	13 (18)	7 (10)
3	53	15 (28)	5 (9)	55	12 (22)	6 (11)	108	27 (25)	11 (10)
4	23	16 (70)	14 (61)	66	24 (36)	17 (26)	89	40 (45)	31 (35)
5	23	16 (70)	12 (52)	24	13 (54)	11 (46)	47	29 (62)	23 (49)

PCa, prostate cancer; CSC, clinically significant prostate cancer.

Any PCa was detected in 35.2% (114/324) and CSC was detected in 23.5% (76/324) of all MRI-guided fusion biopsy targets. The proportion of fusion biopsy targets positive for CSC was similar across the years of the study period (22.0% pre-2018 vs 24.7% 2018-2021). Comparing PI-RADS categories 1-3 to PI-RADS categories 4-5 lesions, the PPV for detecting any PCa was 22.3% (42/188) compared to 52.9% (72/136) respectively. For CSC, the PPV for PI-RADS categories 1-3 and PI-RADS categories 4-5 lesions was 10.1% (19/188) and 41.9% (57/136) respectively.

MRI-guided fusion and systematic 12-core biopsies were concordant for any PCa in 77% of men (141/184) and CSC in 83% (153/184). MRI-guided fusion biopsy detected any PCa in 26/103 (25%) and CSC in 20/131 (15%) of men in whom systematic 12-core biopsy was negative. Systematic 12-core biopsy detected any PCa in 17/94 (18%) and CSC in 11/122 (9%) of men in whom MRI-guided fusion biopsy was negative. In total, 20 patients (11%) had a CSC that would have been missed if MRI-guided fusion biopsy was omitted while 11 (6%) had a CSC that would have been missed without systematic 12-core biopsy.

**Figure 1:**
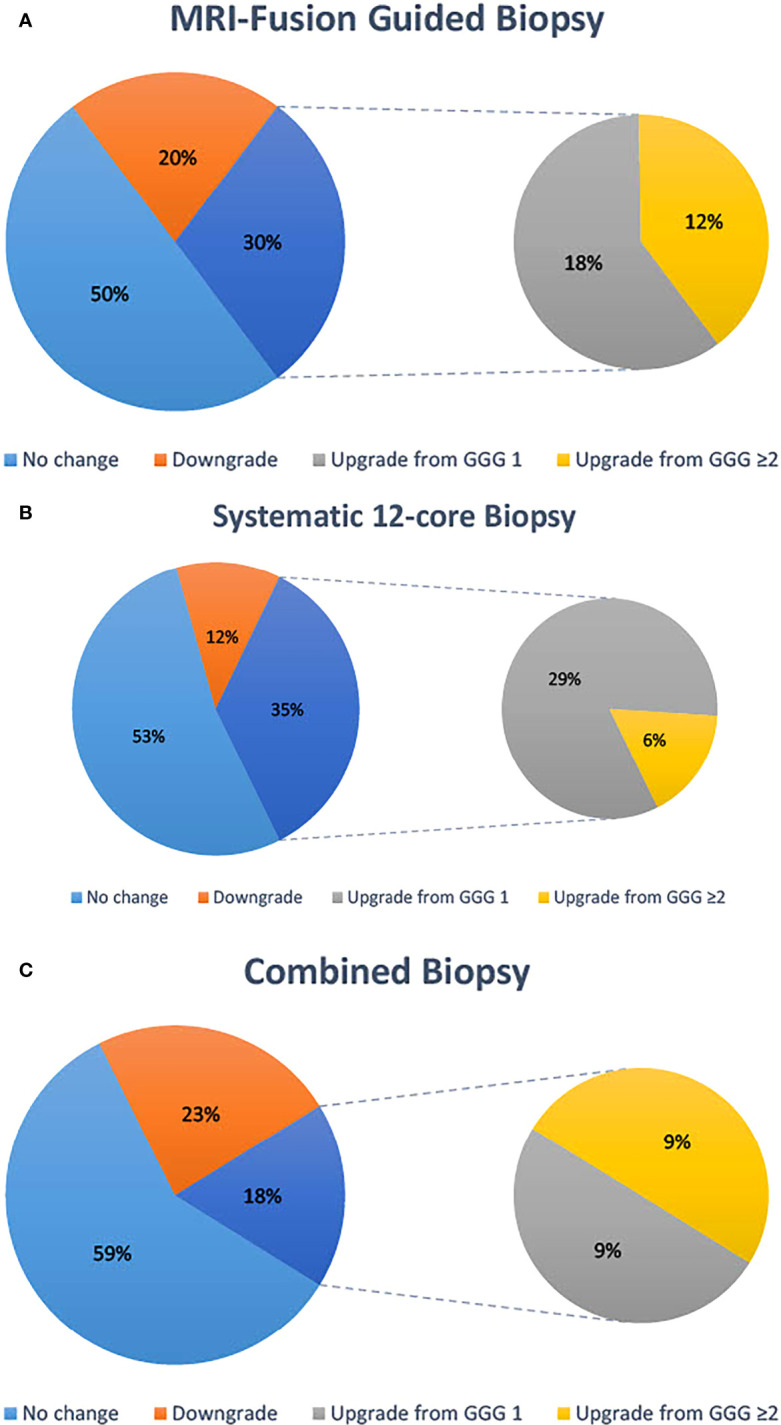
Concordance of highest Gleason score on biopsy with Gleason score reported on prostatectomy specimens. Data are reported for MRI-fusion guided biopsy **(A)**, systematic 12-core biopsy **(B)** and a combined biopsy approach **(C)**.

A total of 40 patients in our cohort ultimately went on receive RP. Six of these were excluded from analysis due to an interval of longer than 1 year from biopsy to prostatectomy leaving 34 patients for analysis. Of these 34 patients, 17 (50%) had no change in GGG between MRI-guided fusion biopsy and prostatectomy specimen, 10 (29%) had their GGG upgraded, and 7 (21%) had their GGG downgraded. Of these, six had an upgrade from non-significant to CSC in the prostatectomy specimen, but none were downgraded from CSC (**Figure 1A**). For systematic 12-core biopsies, 18 (53%) had no change in GGG, 12 (35%) had their GGG upgraded, and 4 (12%) had their GGG downgraded. Of these, 10 had their pathology upgraded to CSC in the prostatectomy specimen and none had their pathology downgraded to clinically insignificant (**Figure 1B**). When considering a combined biopsy approach using the highest Gleason score form either technique, RP resulted in consistent pathology in 20/34 (59%) patients, downgrading GGG in 8 (24%) and upgrading in 6 (18%). Of these, 3 (9%) were upgraded to CSC in the prostatectomy specimen while none were downgraded (**Figure 1C**).

In total, 285/324 (88%) lesions had a recorded lesion diameter on MRI. The median lesion size was 1.35 cm. Of 138 lesions greater than the median, 53 (38.4%) contains any PCa and 38 (27.5%) contained CSC. Of 147 lesions with diameter less than the median, 50 (34.0%) contained any PCa and 32 (21.8%) contained CSC. Among patients with PI-RADS score 4 or 5 lesions, patients with tumor sizes greater than the mean had similar CSC detection rates to patients with smaller lesions, 33/73 (45%) versus 27/68 (40%).

## Discussion

The accurate pathological diagnosis of prostate cancer, and especially clinically significant lesions, remains essential to facilitate appropriate treatment (14–16). Siddiqui et al. found that MRI-guided fusion biopsies preferentially detected high GGG tumors and mitigated the detection of clinically insignificant cancers, a finding which has since been supported by two systematic reviews (17–19). Rastinehad et al. also noted that MRI-guided fusion biopsies are more likely to upgrade GGG 1 lesions detected on systematic 12-core biopsy to GGG 2 or higher (20). Another study randomized patients to MRI-guided fusion biopsy versus systematic 12-core biopsy and found no difference in CSC detection rate but concluded that MRI-guided fusion biopsy was less invasive and could replace systematic 12-core biopsies (21). Additionally, there is evidence supporting the possible superiority of fusion biopsy in the detection of anterior cancer which are often clinically significant (22). However, optimal utilization of systematic 12-core biopsy and MRI-guided fusion biopsy remains an unanswered question and an active area of investigation (12–17). Herein, we attempt to further that literature from the novel of a large community cancer program. Our data are in the setting of a continuous quality improvement protocol which has previously been hypothesized to improve histological data and quality of care (23).

Our experience shows that a combined biopsy approach increases the detection of CSC. This is consistent with the findings of Ahdoot et al (13), and contrary to some earlier studies, supports a continued role for systematic 12-core biopsies (20, 21). Additionally, in patients who underwent RP a combined biopsy approach increased pathologic concordance. Patients with GGG ≤2 had less than half the chance of upgrade to CSC on surgical pathology with both MRI-guided fusion and systematic 12-core biopsy than either alone. This should help mitigate concerns of underdiagnosis of higher Gleason grade prostate cancer. Novel commercially available clinical-genomic tests four kallikrein panel algorithm (4Kpanel), Oncotype DX, Prolaris, and Decipher can also aid decision making for clinicians. For example, these tests have been shown to reclassify men with NCCN low-risk PCa at greater risk for development of metastatic disease (24, 25), identify men with NCCN high-risk PCa who may be candidates for active surveillance (26), and increase confidence for community providers to recommend active surveillance (27). In the future it is possible these tests may help personalize biopsy decisions for many patients. Specifically with the 4Kpanel multiple studies have shown that this test in conjunction with MRI may help reduce unnecessary biopsies (28, 29).

Our experience from a large community-based practice adds to existing literature from clinical trials (17, 21) and academic medical centers (11, 13, 17). With the majority of patients receiving treatment at a community-based practice, our results may be more broadly applicable than existing data. Further, earlier work has described the reproducibility of MRI-guided fusion biopsy results as a potential limitation given concern of technical expertise required in the community setting (13). Our results may help alleviate some of these concerns.

A continuous quality assurance protocol was utilized in which radiologists reviewed discordant MRIs. This process appeared to impact MRI interpretation with fewer low-risk lesions identified and fewer benign lesions biopsied over time. This is clinically meaningful, likely resulting in reduction in toxicity (30), healthcare costs (31), and detection of non-CSC (32) while maintaining sensitivity for CSC.

Lastly, we evaluated the interaction between radiographic target lesion size and likelihood of CSC on biopsy. We found that larger targets were slightly more likely to contain CSC, a finding which appeared to be largely constrained to high PI-RADS score lesions. Maximum tumor diameter in prostate cancer has been an area of recent interest in prostate cancer with studies showing unfavorable outcomes with large tumors (33) as well as recent studies applying increased radiation dose to the dominant nodule (34). While we are not able to evaluate outcomes based on tumor size, our data adds further evidence that larger tumors are more likely to be associated with clinically significant disease.

Our study also has several limitations. MRI-targeted biopsies were performed prior to systematic biopsies leading to the possibility MRI-guided fusion biopsy could influence systematic 12-core biopsy results. Further, there were multiple PI-RADS versions over the course of the study period which may influence results. Our study was also limited to patients with MRI-visible target lesions. Additionally, we are unable to compare the various types of MRI guided biopsy. Various studies have examined the benefits of visual-registration, software assisted registration and in-bore MRI, and have demonstrated some meaningful differences, such as a higher percentage of per-core malignant cells with in-bore techniques (35). However, there remains no consensus on the optimal technique and further work is required to elucidate this (36). RP was not performed in all the patients, which creates the possibility of selection bias in the prostatectomy cohort. Finally, we did not track cancer outcomes and are unable to comment on the impact of factors such as tumor size on survival or disease progression.

## Conclusion

Among patients with MRI-visible prostate lesions, the addition of MRI-guided fusion biopsy to systematic 12-core biopsy increased the detection of CSC. Omission of either biopsy type would have resulted in the underdiagnosis of CSC. Furthermore, among the patients who underwent RP, combined biopsy decreased the rate of upstaging at time of surgery. These findings were in the setting of a continuous quality assurance protocol, which decreased the rate of non-CSC biopsy results. Data contributes the largest experience for MRI-guided fusion biopsies from a community-based oncology setting and support combined utilization of MRI-guided fusion and systematic 12-core biopsies at academic and community-based cancer centers.

## Data Availability Statement

The raw data supporting the conclusions of this article will be made available by the authors, without undue reservation.

## Author Contributions

All authors provided substantial contributions to the conception, research, writing and editing of this manuscript, and all authors have approved the final manuscript for submission.

## Conflict of Interest

The authors declare that the research was conducted in the absence of any commercial or financial relationships that could be construed as a potential conflict of interest.

## Publisher’s Note

All claims expressed in this article are solely those of the authors and do not necessarily represent those of their affiliated organizations, or those of the publisher, the editors and the reviewers. Any product that may be evaluated in this article, or claim that may be made by its manufacturer, is not guaranteed or endorsed by the publisher.
